# Macrolides in Chronic Inflammatory Skin Disorders

**DOI:** 10.1155/2012/159354

**Published:** 2012-05-17

**Authors:** Abdullateef A. Alzolibani, Khaled Zedan

**Affiliations:** ^1^Dermatology Department, College of Medicine, Qassim University, P.O. Box 30109, Buraidah 51477, Saudi Arabia; ^2^Pediatric Department, College of Medicine, Qassim University, P.O. Box 30109, Buraidah 51477, Saudi Arabia

## Abstract

Long-term therapy with the macrolide antibiotic erythromycin was shown to alter the clinical course of diffuse panbronchiolitis in the late 1980s. Since that time, macrolides have been found to have a large number of anti-inflammatory properties in addition to being antimicrobials. These observations provided the rationale for many studies performed to assess the usefulness of macrolides in other inflammatory diseases including skin and hair disorders, such as rosacea, psoriasis, pityriasis rosea, alopecia areata, bullous pemphigoid, and pityriasis lichenoides. This paper summarizes a collection of clinical studies and case reports dealing with the potential benefits of macrolides antibiotics in the treatment of selected dermatoses which have primarily been classified as noninfectious and demonstrating their potential for being disease-modifying agents.

## 1. Introduction

The term “macrolide” encompasses a diverse family of unrelated compounds with large macrolactam rings. The macrolide antibiotics consist of 14-, 15-, and 16-member macrolactam ring antimicrobials. Erythromycin A, the prototype macrolide antibiotic was isolated from a Philippine soil sample in the 1940s and was first marketed in 1952 as an alternative therapy to beta lactam agents for the treatment of infections with Gram-positive cocci. During the 1990s clarithromycin, roxithromycin, and azithromycin were introduced. Macrolide antibiotics inhibit RNA-dependent protein synthesis by reversibly binding to the 50S ribosomal subunit of a susceptible microorganism [1]. 

 Macrolides are widely used to treat infections of soft tissues and of the respiratory tract due to their efficacy against Gram-negative and Gram-positive bacteria, including intracellular germs such as Chlamydia and Legionella [2–4]. They are considered safe and easily tolerable. Their main side effects are nausea, vomiting, diarrhea, and abdominal pain, which become more evident when erythromycin is used in place of the other macrolides [5]. Mounting evidence suggests that macrolide antibiotics have both anti-inflammatory and immune-modulatory properties and are thus beneficial to chronic pulmonary diseases such as diffuse panbronchiolitis, cystic fibrosis, asthma, and bronchiectasis. These properties were suspected upon the realization that erythromycin decreased the need for corticosteroids in asthma treatment [6]. It must be pointed out that immune modulation is the suppression of inflammation and immune hyperactivation without causing immune depression (immunosuppression) [7].

Macrolides antibiotics have been shown to modify host functions apart from the antimicrobial potency. They may directly influence phagocyte and lymphocyte function as well as chemotaxis. Effects on the generation and release of various cytokines involved in the inflammatory process have been studied both *in vivo and in vitro* [8].

Interest in the immunomodulatory effects of macrolides began in the 1960s with the observation that the 14-member antibiotic, troleandomycin, was an effective “steroid-sparing” agent when used to treat patients with severe asthma [9]. It has been more than 20 years since the immunomodulatory effects of macrolides were accepted as a standard of care for the treatment of diffuse panbronchiolitis (DPB) in Japan [10]. Erythromycin and clarithromycin are also widely used in Japan for the therapy of sinusitis and chronic obstructive pulmonary disease (COPD) [11]. In more recent years, azithromycin has been widely adopted as immunomodulatory agents for the treatment of cystic fibrosis (CF) and bronchiectasis.

The anti-inflammatory effects of macrolides are significant. The historical change in the natural course of diffuse panbronchiolitis (DPB), a fatal disorder of the airways, following the introduction of erythromycin in its treatment has focused attention of researchers on the anti-inflammatory properties of macrolides. The clinical impact on diffuse panbronchiolitis (DPB) has improved 10-year survival from 12% to more than 90% for these patients [12]. The immunomodulatory activity of macrolides has been a source of mechanistic research as well as clinical research in non-DPB inflammatory airway disease. Suppression of neutrophilic inflammation of the airways has been demonstrated as the most robust immunomodulatory response from 14- and 15-membered ring macrolides [13].

Macrolide antibiotics are known for their efficacy in treating acute airway infections, but just as importantly, they are also effective anti-inflammatory agents. Their anti-inflammatory properties have been studied most thoroughly in chronic inflammatory airway diseases, particularly diffuse panbronchiolitis (DPB). Erythromycin, azithromycin, clarithromycin, and roxithromycin inhibit chemotaxis and infiltration of neutrophils into the airway and, subsequently, decrease mucus secretion. Mucus formation, a significant cause of morbidity and mortality in patients with chronic airway inflammation, is directly inhibited by macrolides and suppressed by decreased inflammation in the airway. The mechanisms of action for the anti-inflammatory properties of the macrolides are clearly multifactorial. Macrolides inhibit the production of many proinflammatory cytokines, such as interleukin (IL)-1, IL-6, IL-8, and tumor necrosis factor-alpha, perhaps by suppressing the transcription factor nuclear factor-kappa B or activator protein-1. Inhibition of cytokine production has been seen *in vitro* and also in bronchoalveolar lavage fluid, which contains less IL-8 and fewer neutrophils after treatment with macrolides. Macrolides also inhibit formation of leukotriene B4, which attracts neutrophils, and inhibit the release of superoxide anion by neutrophils that may be present in the airway. An important aspect of inflammation is extravasation of neutrophils into the tissues. Macrolides block formation of adhesion molecules necessary for neutrophil migration. Together, these anti-inflammatory effects result in improved pulmonary functions and fewer airway infections. In patients with DPB, the anti-inflammatory effects lead to a significant increase in survival. These effects might be pharmacological functions of the macrolide itself, independent of antibiotic effects. Apart from antibacterial effects, macrolides have effects on neutrophil function (decreased oxidant production, apoptosis) and on the production of cytokines involved in the inflammation cascade (decreased production of IL-1, IL-6, IL-8, and TNF and increased production of IL-10 and, possibly, IL-4). With regard to T lymphocytes, erythromycin (EM) and its derivatives inhibit T-lymphocyte proliferation and induce T-lymphocyte apoptosis [14, 15].

In this paper, we present a collection of clinical studies and case reports dealing with the potential benefits of macrolides antibiotics in the treatment of selected dermatoses which have primarily been classified as noninfectious. A comprehensive search in the PubMed/MEDLINE and Embase databases was performed. We examined the eligible literature. Studies that dealt with the effects of macrolides as anti-inflammatory and immune-modulator in skin and hair disorders were included.

**(A) Macrolides and Intractable Rosacea**

Rosacea is a common cutaneous disorder which occurs most frequently in light-skinned, middle-aged women. There are variable cutaneous signs of rosacea such as flushing, erythema, telangiectasia, edema, papules, and pustules [16].

Conventional treatment of rosacea is based on a combination of systemic and topical antibiotics. Since the 1950s, tetracycline and erythromycin are the most commonly used oral antibiotics [17]. The therapeutic activity of commonly used antimicrobials including tetracycline, doxycycline has been mainly attributed to their anti-inflammatory activities [18]. However, long-term treatment with antibiotics is not well tolerated due to requiring frequent administration, poor compliances and side effects including gastrointestinal intolerance, photosensitivity, and candidiasis [19].

Azithromycin is effective in treating rosacea. Facial skin biopsies were taken from 17 subjects with papulopustular rosacea and 25 healthy controls. Rosacea patients had greater skin reactive oxygen species levels than healthy controls (*P* < 0.001). Rosacea subjects then received oral azithromycin 500 mg on three days each week for 4 weeks. A statistically significant decrease in chemiluminescence, a measurement of the generation of reactive oxygen species, was demonstrated after treatment with azithromycin [19].

The utility of oral azithromycin was confirmed by several other clinical studies. Fernandez-Obregon [20] reported that all of ten patients who were not tolerated or controlled by conventional treatment of rosacea demonstrated a significant improvement with the oral use of azithromycin. In addition, Modi et al. [21] treated a 67-year-old man who had photosensitivity to the doxycycline and hyperpigmented dyschromia to the minocycline with an oral use of azithromycin in a dose of 250 mg 3 times weekly. Bakar et al. [19] reported that treatment with oral azithromycin led to 75% decreases in the total number of lesions and an 89% decrease in inflammatory lesions compared with basal status. Another open-label study showed that azithromycin is as effective as standard dose of doxycycline and has a positive impact on the quality of life of patients compared with conventional treatment regimens [22].

Kim et al. [23] treated a 52-year-old woman who had intractable rosacea not responding to various conventional treatments including topical benzoyl peroxide and metronidazole as well as oral metronidazole, isotretinoin, and doxycycline, by using oral azithromycin 500 mg per day for 2 weeks. The authors reported that the lesions had mostly disappeared, and no specific side effects related to the azithromycin were noted.

**(B) Macrolides and Adult-Onset Still's Disease (AOSD)**

Adult-onset Still's disease (AOSD), an autoinflammatory syndrome of unknown etiology, typically manifests with spiking fevers, polyarthritis, and characteristic evanescent rash. Thanou-Stavraki et al. [24] described a young woman with AOSD complicated by calf fasciitis that serendipitously responded to clarithromycin administered for another indication. Remarkable improvement followed rechallenges with clarithromycin for subsequent AOSD flares. Although AOSD pathogenesis remains unclear, a role for dysregulation of innate immunity is suggested. Based on this possible innate immune mechanism, the investigators suspected that macrolides may have induced a therapeutic response in this patient with AOSD.

Saviola et al. [25] treated six cases of AOSD with clarithromycin (CM) in combination with low-mild dose of glucocorticoids (GC), and methotrexate (MTX). Four of them were not responsive to high-dose GC added to disease-modifying antirheumatic drugs (DMARDs), while two of them were treated with low-mild dose of GC added to CM from the beginning. CM, 500 mg b.i.d., was added to a mild-low dose of GC and to MTX. The dose of the drugs was reduced (and stopped where possible) following clinical and laboratory parameters. ACR criteria were used to assess clinical improvement. At 6 months, 5 patients reached ACR 70% and could stop any therapy in 6–18 months; 1 continued chronic therapy with low-dose GC added to CM and MTX to maintain ACR 50%. The authors reported that CM can be a useful drug for the treatment of AOSD, even in patients not responsive to high-dose GC and DMARDs.

**(C) Macrolides and SAPHO Syndrome**

In 1987, synovitis, acne, pustulosis, hyperostosis, and osteitis (SAPHO) syndrome was proposed as an umbrella term for a group of diseases with similar musculoskeletal manifestations, in particular hyperostosis of anterior chest wall, synovitis, and multifocal aseptic osteomyelitis, observed in association with dermatologic conditions such as palmo-plantar pustulosis, severe acne, and hidradenitis suppurativa [26]. Despite recent advances in the understanding of the epidemiologic, pathophysiologic, and immunogenetic mechanisms involved in SAPHO syndrome, etiopathogenesis remains poorly understood. Propionibacterium acnes, the microorganism associated with acne, has been recovered on bone biopsy in some patients, but the possible pathogenetic role of an infectious agent in a genetically predisposed individual, resulting in exaggerated inflammatory response as “reactive osteitis,” is a largely unproven hypothesis [27].

Schaeverbeke and colleagues reported one case of successful treatment of a SAPHO patient with azithromycin [28]. Kirchhoff and colleagues presented data for seven patients being treated successfully with azithromycin over 5 months [29]. Assmann et al. [30] reported successful control of the disease with azithromycin over 16 weeks. After antibiotic discontinuation, however, disease relapse was observed.

Matzaroglou et al. [31] reported five patients with SAPHO syndrome (3 women; 2 men), ages 27 to 44 years, showed remarkable response to treatment with macrolide antibiotic (clindamycin) and nonsteroid anti-inflammatory drugs (lornoxicam). All patients did well and remained symptom-free for up to four years, after a 3–8-month course of treatment. The authors concluded that appropriate therapy with antibiotics and NSAIDs can produce rapid symptom resolution, while avoiding unnecessary procedures and long-term antibiotic therapy.

**(D) Macrolides and Psoriasis**

Psoriasis is a well-known clinical description of an inflammatory skin disorder with other manifestations of what, until now, has been considered as a single disease entity. The characteristic skin lesion is persistent, erythematous, indurated and scaly, reflecting infiltration of inflammatory cells and increased proliferation and turnover of keratinocytes. The infiltrates in the dermis and the deeper layer of the epidermis mostly comprise of macrophages and T cells. Stimulation of dendritic cells and macrophages, which are called antigen-presenting cells, results in the activation of T-helper (Th) cells. These differentiate into IFN-gamma, producing Th 1 cells, and IL-17, producing Th 17 cells. Interaction of these cells with macrophages, mast cells, and neutrophils results in cytokine release and inflammation, leading to keratinocyte proliferation [32].

Psoriasis is characterized by the presence of neutrophil overactivation and overproduction of interleukin (IL)-6 and IL-8 from keratinocytes [33]. Macrolide antibiotics are widely used as antimicrobial agents. It is now clear that macrolide antibiotics inhibit the production of many proinflammatory cytokines, such as IL-6, IL-8, and tumor necrosis factor (TNF)-*α*, perhaps by suppressing the transcription factors nuclear factor (NF)-*κ*B or activator protein-1, and reduce neutrophil activity [34]. There are conflicting views in the literature regarding the efficacy of macrolides on psoriasis. Although in some studies it has been reported that intervention by antibiotics is not beneficial [35, 36], other studies have shown efficacy of macrolides in psoriasis [33].

A high incidence of streptococcal throat infection as the main trigger for psoriasis exacerbations favors streptococcal antigens as a causative agent, which may induce cross-reactive T-cell responses against skin components [37, 38]. Staphylococcal superantigens have also been associated with psoriatic disease [31].

Ohshima et al. [39] deserves special attention, where ten patients with chronic plaque psoriasis were enrolled and advised to take 150 mg Roxithromycin (a macrolide) orally twice daily for 1 to 7 weeks. Six out of the ten patients exhibited a decrease in psoriasis area and severity index (PASI) score. The mechanism by which macrolides downregulates the host inflammatory response was unclear but certainly multifactorial.

Macrolides, as a class, and azithromycin in particular, have a characteristic immunomodulatory and anti-inflammatory potential, in addition to their main antibacterial action against streptococci. Suppression of secretions of the cytokine-interleukin (IL)-8 may be important. This macrolide probably also suppresses immunological events in interferon gamma-treated keratinocytes, including expression of MHC class II, secretion of IL-1 alpha, and superantigen presenting ability [40, 41].

Saxena and Dogra [42] tried oral azithromycin in a single blind randomized case-control trial. 50 patients with moderate-to-severe chronic plaque psoriasis were enrolled. Of these, 30 randomly selected patients received azithromycin for 48 weeks as a single oral 500 mg daily dose for 4 days with a gap of 10 days (total 24 such courses). The remaining 20 patients received a vitamin C tablet (nonchewable) in the same dosage schedule. A significant improvement in PASI score was noted from 12 weeks in the majority of patients in the azithromycin group. At the end of 48 weeks, 18 patients (60%) showed excellent improvement, while 6 patients (20%) showed good improvement, and 4 patients (13.33%) showed mild improvement. A significant improvement in the skin lesions was noted at 12 weeks of azithromycin therapy. Based on this study, the authors reported that the results substantiated the hypothesis that chronic ongoing stimulus by the streptococci or its superantigen was indispensable in maintaining the disease.

17 subjects participated in an open trial of macrolides for treatment of psoriasis. Mean PASI scores dropped significantly, and itch was reduced in 11 subjects after therapy. This study showed that macrolide antibiotics may be effective for treatment of psoriatic skin lesion, and that they may have antipruritic effects [33].

Tamaki investigated the antipruritic effects of macrolide antibiotics in several pruritic skin diseases. They found that in most of the patients, the drug was very effective. The reason for the antipruritic effect is not known; however, it is suggested possibly that macrolide antibiotics inhibit production of cytokines or neuropeptides that cause pruritus [43].

Polat et al. [44] studied patients with psoriasis. The patients were divided into two treatment groups: one to receive erythromycin and topical steroids and the other only topical steroids: the first group were treated with erythromycin 1000 mg/day and topical corticosteroids for 4 weeks, while the control group were treated only with topical corticosteroids. The study group comprised 36 patients; the control group comprised 24 patients. There was no significant difference between the baseline mean Psoriasis Area and Severity Index (PASI) of the two groups. They reported that the treatment used for the study group was more effective against pruritus than that used for the control group. Six patients with severe pruritus and six patients with moderate pruritus in the study group found that itch disappeared completely after the treatment. In the control group, none of the patients with severe or moderate pruritus found that itch disappeared completely.

**(E) Macrolides and Alopecia Areata, Associated with *H. pylori* Infection**

Campuzano-Maya [45] described a case of a 43-year-old man with patchy alopecia areata and *H. pylori* infection; the patient had hair regrowth after bacterial eradication. The patient was prescribed first-line *H. pylori* eradication with proton pump inhibitor (omeprazole) 20 mg twice daily, amoxicillin 1000 mg twice daily, and clarithromycin 500 mg twice daily for 14 days and was followed photographically every 2 wks. He was instructed not to take or apply any medications for alopecia areata. The patient's condition started to improve within 4 wks of completing *H. pylori* eradication. By week 16, the patient had completely reversed the hair loss, and by week 44, he remained *H. pylori*-negative and completely cured of alopecia areata. The author reported that this is the first documented case of reversed hair loss after *H. pylori* eradication and, if such an association is confirmed by epidemiological studies designed for this purpose, new therapeutic options could be available for these patients, especially in areas where infection with *H. pylori* is highly prevalent.

**(F) Macrolides and Chronic Urticaria, Associated with **H. pylori **Infection**

Chronic urticaria is one of the most frequent skin diseases in medical practice. Urticaria is defined as acute if the whealing persists for less than six weeks and as chronic if it persists for longer. Chronic urticaria that lasts from several years to decades significantly impairs the quality of life. There is evidence that *Helicobacter pylori* has a critical role in different extragastric diseases such as chronic urticaria. Ben Mahmoud et al. [46] presented a case of chronic urticaria in an adult patient with *H. pylori* infection and disease regression after triple anti-*H. pylori* therapy. In contrast to the autoimmune mechanisms involved in chronic urticaria against which no specific treatment strategy has been developed, infections with *H. pylori* could be treated with triple therapy. The authors suggested that laboratory tests for the detection of this pathogen should be performed in patients with chronic urticaria.

**(G) Macrolides and Pityriasis Rosea**

Sharma et al. [47] performed a clinical study to evaluate the efficacy of erythromycin in patients with pityriasis rosea (PR). Ninety patients over a period of 2 years were alternatively assigned to treatment group or placebo group. Patients in the treatment group received erythromycin in divided doses for 14 days. The response was categorized as complete response, partial response, or no response. Complete response was observed in 33 patients (73.33%) in the treatment group and none in the placebo group. The authors concluded that oral erythromycin was effective in treating patients with pityriasis rosea, and that the effect of erythromycin may be related to its anti-inflammatory properties.

Rasi et al. [48] conducted a placebo-controlled study on 184 patients with pityriasis rosea attending the outpatient dermatology department clinic. Adult patients were treated with 200 mg of erythromycin 4 times daily, and children were treated with 20 to 40 mg/kg daily in 4 divided doses. Controls were given a placebo (an emollient cream) that was not identical in appearance. Subjects were seen at follow-up visits 2, 4, 6, and 8 weeks after starting treatment. Both groups were comparable with regard to sex, age, and mean duration of disease at the time of attending the clinic. They found no significant difference between the 2 treatment groups at weeks 4, 6, and 8 after beginning of treatment.

Other authors believe that the use of macrolides is best considered experimental and should not be adopted into routine clinical practice until further studies are conducted and results are published. Even if macrolides are finally proven to be effective in modifying the course of PR, this does not substantiate that PR is caused by a bacterial rather than a viral infection. Macrolides have anti-inflammatory and immunomodulating effects that might affect the course of PR or other cutaneous eruptions independent of their antibacterial properties [49].

**(H) Macrolides and Pityriasis Lichenoides**

Pityriasis lichenoides is an uncommon reactive papulosquamous eruption of unknown origin. Truhan et al. [50] performed a study to determine the effects of erythromycin in pityriasis lichenoides. Fifteen of twenty-two children with pityriasis lichenoides were treated with oral erythromycin. Eleven (73%) had a remission, usually within 2 months. Two others showed partial improvement, and two were unimproved. Seven of the children who experienced a remission were off erythromycin and free of lesions after 2 to 5 months of therapy. The authors concluded that a trial of erythromycin should be considered in children with pityriasis lichenoides before other, possibly more toxic, measures are instituted.

Skinner and Levy [51] reported two cases of persistent pityriasis lichenoides et varioliformis acuta (PLEVA) unresponsive to tetracycline and erythromycin that rapidly resolved with bimonthly treatment with azithromycin for 5 days. The first case was a 51-year-old female started on azithromycin 500 mg on day 1 and 250 mg on days 2 through 5, to be taken on the first and third weeks of the month. One week after starting the first course, she reported that no new lesions had formed, and that the current lesions were resolving. After 3 weeks and two courses of azithromycin, the patient was clear of all lesions. She has remained clear for 6 months. The second case was a 5-year-old boy in whom erythromycin taken for 3 months did not improve the rash. He was then started on the same azithromycin regimen stated above. Eight weeks later, the patient had completed four courses of azithromycin. He had marked improvement, with only a few remaining smooth papules. He was continued on azithromycin for one more course and was clear of all lesions on 1-month followup and again 2 months later.

**(I) Macrolides and Bullous Pemphigoid**

Bullous pemphigoid is the most common autoimmune-mediated bullous disease in men. Mensing and Krausse [52] tested erythromycin combined with a low-dose methylprednisolone in eleven patients in a prospective study. A historical collective of the last 33 patients treated before this study was started served as the control group. The duration of hospitalization as an expression of therapeutic response, but also of lowered side effects dropped down from 43 to 33 days in the erythromycin treated group. Altomare et al. [53] reported that the macrolide antibiotic erythromycin has been effective in bullous pemphigoid in their studied patients. Fox et al. [54] reported two patients with bullous pemphigoid treated with erythromycin demonstrated improvement.

**(J) Successful Treatment of Idiopathic Thrombocytopenic Purpura with Macrolides**

Ohe and Hashino [55] reported 3 cases of primary immune thrombocytopenia (ITP) patients who were successfully treated with macrolides, irrespective of *Helicobacter pylori *(*H. pylori*) infection status. Case  1, an 88-year-old woman who was an *H. pylori*-positive ITP patient, was treated with clarithromycin (CAM). CAM was effective temporarily. As an alternative to CAM, she was successfully treated with erythromycin (EM) for more than 7 months. Case  2, a 61-year-old man who was an *H. pylori*-negative ITP patient, was unsuccessfully treated with CAM but successfully treated with EM. Case  3, a 75-year-old woman who was a *H. pylori*-negative ITP patient, was treated with CAM. CAM was effective temporarily. After approximately 6 months, she was treated with EM for a common cold, and her platelet count increased rapidly. The authors concluded, based on these findings, that macrolide treatment was effective for ITP. The effectiveness of macrolides might suggest immunomodulatory effects as well as antibacterial effects for *H. pylori*.

In a previous work, the authors have already reported 3 cases of idiopathic thrombocytopenic purpura (ITP), also known as primary immune thrombocytopenia, which show increased platelet counts following clarithromycin treatment, irrespective of *H. pylori* infection status [56].

The authors attributed this therapeutic success of macrolides in treating cases of ITP to the immunomodulatory effects of macrolides. Immunomodulatory effects from macrolide antibiotics might be obtained by the eradication of bacteria or by modulation of the immune system involving the mucosa on which commensal bacteria reside [57].

## 2. Conclusion

Despite the small number of studies shedding light on the anti-inflammatory and immunomodulatory mechanisms of the macrolides, there is strong evidence providing support to the benefit of using this type of drug for the long term and in low doses to treat some chronic inflammatory skin disorders. The macrolides have some potentially useful immunomodulatory effects. Although additional studies are needed, macrolide therapy in some of chronic dermatoses has the potential of modifying the morbidity and possibly ameliorating the severity of some, but not all, of these conditions. Further well-designed, adequately powered randomized controlled trials are required.

## References

[1] Washington JA, Wilson WR (1985). Erythromycin: a microbial and clinical perspective after 30 years of clinical use. *Mayo Clinic Proceedings*.

[2] López-Boado YS, Rubin BK (2008). Macrolides as immunomodulatory medications for the therapy of chronic lung diseases. *Current Opinion in Pharmacology*.

[3] Healy DP (2007). Macrolide immunomodulation of chronic respiratory diseases. *Current Infectious Disease Reports*.

[4] Florescu DF, Murphy PJ, Kalil AC (2009). Effects of prolonged use of azithromycin in patients with cystic fibrosis: a meta-analysis. *Pulmonary Pharmacology and Therapeutics*.

[5] Whitman MS, Tunkel AR (1992). Azithromycin and clarithromycin: overview and comparison with erythromycin. *Infection Control and Hospital Epidemiology*.

[6] Itkin IH, Menzel ML (1970). The use of macrolide antibiotic substances in the treatment of asthma. *Journal of Allergy*.

[7] Shinkai M, Rubin BK (2005). Macrolides and airway inflammation in children. *Paediatric Respiratory Reviews*.

[8] Brinkmeier T, Frosch PJ (2002). Oral antibiotics with antiinflammatory/immunomodulatory effects in the treatment of various dermatoses. *Hautarzt*.

[9] Spector SL, Katz FH, Farr RS (1974). Troleandomycin: effectiveness in steroid dependent asthma and bronchitis. *Journal of Allergy and Clinical Immunology*.

[10] Kudoh S, Uetake T, Hagiwara K, Hirayama M, Hus LH, Kimura H (1987). Clinical effects of low-dose long-term erythromycin chemotherapy on diffuse panbronchiolitis. *Nihon Kyobu Shikkan Gakkai Zasshi*.

[11] Tamaoki J, Takeyama K, Tagaya E, Konno K (1995). Effect of clarithromycin on sputum production and its rheological properties in chronic respiratory tract infections. *Antimicrobial Agents and Chemotherapy*.

[12] Kudoh S, Azuma A, Yamamoto M, Izumi T, Ando M (1998). Improvement of survival in patients with diffuse panbronchiolitis treated with low-dose erythromycin. *American Journal of Respiratory and Critical Care Medicine*.

[13] Umeki S (1993). Anti-inflammatory action of erythromycin: its inhibitory effect on neutrophil NADPH oxidase activity. *Chest*.

[14] Labro MT (1998). Anti-inflammatory activity of macrolides: a new therapeutic potential?. *Journal of Antimicrobial Chemotherapy*.

[15] Wu L, Zhang W, Tian L, Bao K, Li P, Lin J (2007). Immunomodulatory effects of erythromycin and its derivatives on human T-lymphocyte in vitro. *Immunopharmacology and Immunotoxicology*.

[16] James WD, Berger TG, Elston DM (2006). *Andrew's Diseases of the Skin*.

[17] Del Rosso JQ (2000). Systemic therapy for rosacea: focus on oral antibiotic therapy and safety. *Cutis*.

[18] Humbert P, Treffel P, Chapuis JF, Buchet S, Derancourt C, Agache P (1991). The tetracyclines in dermatology. *Journal of the American Academy of Dermatology*.

[19] Bakar O, Demirçay Z, Yuksel M, Haklar G, Sanisoglu Y (2007). The effect of azithromycin on reactive oxygen species in rosacea. *Clinical and Experimental Dermatology*.

[20] Fernandez-Obregon A (2004). Oral use of azithromycin for the treatment of acne rosacea. *Archives of Dermatology*.

[21] Modi S, Harting M, Rosen T (2008). Azithromycin as an alternative rosacea therapy when tetracyclines prove problematic. *Journal of Drugs in Dermatology*.

[22] Akhyani M, Ehsani AH, Ghiasi M, Jafari AK (2008). Comparison of efficacy of azithromycin vs. doxycycline in the treatment of rosacea: a randomized open clinical trial. *International Journal of Dermatology*.

[23] Kim JH, Oh YS, Choi EH (2011). Oral azithromycin for treatment of intractable rosacea. *Journal of Korean Medical Science*.

[24] Thanou-Stavraki A, aberle T, Aksentijevich I, Bane BL, Harley JB (2011 ). Clarithromycin in adult-onset still's disease: a potentially useful therapeutic. *Journal of Clinical Rheumatology*.

[25] Saviola G, Benucci M, Abdi-Ali L (2010). Clarithromycin in adult-onset Still’s disease: a study of 6 cases. *Rheumatology International*.

[26] Chamot AM, Benhamou CL, Kahn MF, Beraneck L, Kaplan G, Prost A (1987). Acne- pustulosis- hyperostosis- osteitis syndrome. Results of a national survey: 85 cases. *Revue du Rhumatisme et des Maladies Osteo-Articulaires*.

[27] Colina M, Lo Monaco A, Khodeir M, Trotta F (2007). Propionibacterium acnes and SAPHO syndrome: a case report and literature review. *Clinical and Experimental Rheumatology*.

[28] Schaeverbeke T, Lequen L, de Barbeyrac B (1998). Propionibacterium acnes isolated from synovial tissue and fluid in a patient with oligoarthritis associated with acne and pustulosis. *Arthritis & Rheumatism*.

[29] Kirchhoff T, Merkesdal S, Rosenthal H (2003). Diagnostic management of patients with SAPHO syndrome: use of MR imaging to guide bone biopsy at CT for microbiological and histological work-up. *European Radiology*.

[30] Assmann G, Kueck O, Kirchhoff T (2009). Efficacy of antibiotic therapy for SAPHO syndrome is lost after its discontinuation: an interventional study. *Arthritis Research and Therapy*.

[31] Matzaroglou C, Velissaris D, Karageorgos A, Marangos M, Panagiotopoulos E, Karanikolas M (2009). SAPHO syndrome diagnosis and treatment: report of five cases and review of the literature. *The Open Orthopaedics Journal*.

[32] Balci DD, Duran N, Ozer B, Gunessacar R, Onlen Y, Yenin JZ (2009). High prevalence of Staphylococcus aureus cultivation and super-antigen production in patients with psoriasis. *European Journal of Dermatology*.

[33] Komine M, Tamaki K (2000). An open trial of oral macrolide treatment for psoriasis vulgaris. *Journal of Dermatology*.

[34] Tamaoki J, Kadota J, Takizawa H (2004). Clinical implications of the immunomodulatory effects of macrolides. *The American Journal of Medicine*.

[35] Owen CM, Chalmers RJG, O’Sullivan T, Griffiths CEM (2001). A systematic review of antistreptococcal interventions for guttate and chronic plaque psoriasis. *British Journal of Dermatology*.

[36] Wilson JK, Al-Suwaidan SN, Krowchuk D, Feldman SR (2003). Treatment of psoriasis in children: is there a role for antibiotic therapy and tonsillectomy?. *Pediatric Dermatology*.

[37] Valdimarsson H, Sigmundsdottir H, Jonsdottir I (1997). Is psoriasis induced by streptococcal super antigens and maintained by M- protein specific T cells that cross react with keratin?. *Clinical & Experimental Immunology*.

[38] Prinz JC (2001). Psoriasis vulgaris- a sterile anti-bacterial skin reaction mediated by cross reactive T cells? An immunological view of the patho physiology of psoriasis. *Clinical and Experimental Dermatology*.

[39] Ohshima A, Takigawa M, Tokura Y (2001). CD8^+^ cell changes in psoriasis associated with roxithromycin-induced clinical improvement. *European Journal of Dermatology*.

[40] Konno S, Adachi M, Asano K, Okamoto K, Takahashi T (1992). Inhibition of human T-lymphocyte activation by macrolide antibiotic, roxithromycin. *Life Sciences*.

[41] Wakita H, Tokura Y, Furukawa F, Takigawa M (1996). The macrolide antibiotic, roxithromycin suppresses IFN-*γ*-mediated immunological functions of cultured normal human keratinocytes. *Biological and Pharmaceutical Bulletin*.

[42] Saxena VN, Dogra J (2010). Long-term oral azithromycin in chronic plaque psoriasis: a controlled trial. *European Journal of Dermatology*.

[43] Tamaki K (2000). Antipruritic effect of macrolide antibiotics. *Journal of Dermatology*.

[44] Polat M, Lenk N, Yalcin B (2007). Efficacy of erythromycin for psoriasis vulgaris. *Clinical and Experimental Dermatology*.

[45] Campuzano-Maya G (2011). Cure of alopecia areata after eradication of helicobacter pylori: a new association. *World Journal of Gastroenterology*.

[46] Ben Mahmoud L, Ghozzi H, Hakim A, Sahnoun Z, Zeghal K (2011). Helicobacter pylori associated with chronic urticaria. *Journal of Infection in Developing Countries*.

[47] Sharma PK, Yadav TP, Gautam RK, Taneja N, Satyanarayana L (2000). Erythromycin pityriasis rosea: a double-blind, placebo-controlled clinical trial. *Journal of the American Academy of Dermatology*.

[48] Rasi A, Tajziehchi L, Savabi-Nasab S (2008). Oral erythromycin is ineffective in the treatment of pityriasis rosea. *Journal of Drugs in Dermatology*.

[49] Chuh A, Lee A, Zawar V, Sciallia G, Kempf W (2005). Pityriasis rosea—an update. *Indian Journal of Dermatology, Venereology and Leprology*.

[50] Truhan AP, Hebert AA, Esterly NB (1986). Pityriasis lichenoides in children: therapeutic response to erythromycin. *Journal of the American Academy of Dermatology*.

[51] Skinner RB, Levy AL (2008). Rapid resolution of pityriasis lichenoides etvarioliformis acuta with azithromycin. *Journal of the American Academy of Dermatology*.

[52] Mensing H, Krausse S (1990). Erythromycin treatment for bullous pemphigoid. *Medizinische Klinik*.

[53] Altomare G, Capella GL, Fracchiolla C, Frigerio E (1999). Treatment of bullous pemphigoid with erythromycin: a reappraisal. *European Journal of Dermatology*.

[54] Fox BJ, Odom RB, Findlay RF (1982). Erythromycin therapy in bullous pemphigoid: possible anti-inflammatory effects. *Journal of the American Academy of Dermatology*.

[55] Ohe M, Hashino S (2011). Successful treatment with erythromycin for idiopathic thrombocytopenic purpura. *Korean Journal of Hematology*.

[56] Ohe M, Kohno M (2003). Three cases of idiopathic thrombocytopenic purpura showing an increase in the platelet count following clarithromycin treatment. *Rinsho Ketsueki*.

[57] Tlaskalova-Hogenova H, Stepankova R, Hudcovic T (2004). Commensal bacteria (normal microflora), mucosal immunity and chronic inflammatory and autoimmune diseases. *Immunology Letters*.

